# Editors introduction: biobanks as sites of bio-objectification

**DOI:** 10.1186/s40504-018-0070-5

**Published:** 2018-02-21

**Authors:** Neil Stephens, Nik Brown, Conor Douglas

**Affiliations:** 1 Brunel University London, London, UK; 2 University of York, York, UK; 3 Maastricht University, Maastricht, The Netherlands

Biobanks and biorepositories have become increasingly important and prevalent since the 1990s as holders and distributors of biological material. They exhibit significant diversity in form and function, from the very small to the very large, from the very specialised to the much more generic, holding collections of diseased and healthy resources, from human, animal and plant, and span private, public and third sectors. They also operate as key mediators in relationships between patients, researchers, regulators and companies as they hold and distribute tissue, data and social credibility. Furthermore, they remain active sites in the mediation of controversy, sometimes causing controversy, sometimes closing controversy. In doing, they become important nodal points of regulatory practice (Douglas et al. 2012; Hansen and Metzler 2012). Their proliferation has resulted in new and dynamic ethical and policy issues in need of critical engagement, some of which are addressed in this thematic issue. A growing literature exists addressing these important issues and opening new ones for inspection. Here we present a set of papers that contribute to this work. The distinctiveness of this thematic issue is the application of a unified theoretical approach.

The thematic issue takes biobanks and biorepositories as empirical and conceptual sites for articulating and applying the theoretical tool kit of Bio-objects, Bio-objectification and Bio-identification (cf. Tupasela and Stephens 2013). The thematic issue builds upon the work of the ISCH COST Action IS1001 “Bio-objects and their boundaries” to offer a set of inter-related papers that retain analytical continuity while exploring different empirical configurations of biobanking practice (cf. Vermeulen et al. 2012). The authors represented here understand the contents of biobanks as bio-objects: referring to “a socially potent biotechnological entity which generates controversy due to its potential challenging of established classifications” (Webster A: Bio-objectification: definitions and tools unpublished internal document. COST Bio-objects action, unpublished). They seek to analyse the role of biobanks in determining the boundaries of bio-objects, through the examination of the active process of ‘bio-objectification’, meaning the process through which different types of bio-socio-technological categorizations contribute to the making of bio-objects. As a consequence of these novel relations, the boundaries between human and animal, organic and nonorganic, living and the suspension of living (and the meaning of death itself), are questioned and destabilized, as new relationships are formed (Tamminen and Vermeulen 2012). The analyst’s task is to map how bio-objects are formed through bio-objectification processes, and to analyse the standardization, stabilization and labelling of a new entity.

Webster (A: Bio-objectification: definitions and tools unpublished internal document. COST Bio-objects action, unpublished) develops a bio-objects conceptual ‘toolkit’ that entails four core dimensions that can underpin analysis using the approach. They are:

1. Matter out of place and the process of bio-objectification: challenging classifications.
2. Controversy in one or more arenas of society: challenging social order.
3. Organizational and institutional processes: Technologies and labour involved, that are sometimes themselves problematized – challenging ‘discovery’.
4. Bio-identification: Consequences on individual, symbolic and structural levels; changing social relations, the emergence of new categories and identities – challenging social relations.

(adapted from Webster, unpublished, p2).

Further building the perspective into a researcher-ready framework, Holmberg et al. (2011), articulate three methodological implications of the bio-objects approach:

1. Mapping and tracking: follow the bio-object as it engages (circulates across) current legal provisions and regulation – understanding the bio-objectification process and its different dimensions.
2. Comparing: determining the divergences and convergences of bio-objects/objectification across different domains.
3. Modeling: identifying stabilizing (shape-holding) and disruptive patterns and processes associated with bio-objectification.

(adapted from Holmberg et al. 2011).

The theoretical framework, and the associated methodological approach, gives a handle to an object’s form as ordered both socially (as embedded within contexts) and materially (as internal to its structure) and the relational processes between the two. These relations can be inspected empirically, and have been used fruitfully to understand bio-objects including microRNA (Chrupek et al. 2012), stem cells (Eriksson 2012), and clinical research patients (Douglas 2012). The five papers in this thematic issue use the framework to better understand biobanking, with core questions addressed including: How is the entity produced as a bio-object in biobanking contexts? Does this entity challenge boundaries/classifications and what boundaries are being challenged? How are the boundaries negotiated? By whom? With what means? At what sites? Are controversies created as a consequence of the challenging of boundaries, and how are controversies being silenced? What kind of labour/practices are involved in the biobanking process? What type of labour is required? What are the organisational frameworks in the Biobanking field? What networks are involved in bio-objectification and in the work of bio-identification? How are identities – of institutions and tissue - stabilised and legitimised? When is ‘bio-identification’ successfully achieved, referring to a ‘regulatable’ and usable status as technical object? We now address each paper in turn.

Tupasela et al. (2015) discuss how populations are constructed through the practices and standards of biobanks. The work is based upon empirical analysis of biobanks based in Canada, Finland, Iceland, Spain, the UK and the USA over two years that collated interviews with biobank representatives, policy makers and regulators as well as documentary evidence. The key focus is the diverse range of engagement strategies employed by various banks and that, through a process of bio-objectification, specific political systems, societies, nationalities, communities, and patient groups are represented and understood to have distinct characteristics. They show, as examples, that the history of nationalistic disputes in Spain leads Spanish biobankers to employ a nuanced approach to constructing what population means and that this is evident in the modes of engagement they participate in, whereas in Canada the view that biobanks are a public good has led to a public constructed as positive and willing to participate, even if engagement practice does not always maximise this potential to its fullest. Importantly, this allows Tupasela, Snell and Cañada to argue that biobanks define populations both through how they analyse their biological characteristics and through how they engage with them.

Boeckhout and Douglas (2015) explore Dutch perspectives on governing the research-care divide in clinical biobanking. The paper reports interviews and observations of Dutch biobanking infrastructures from 2008 to 2013, including the largescale Parelsnoer Instituut. Their key focus is how bio-objectification processes come into being, and the capacity they have to redefine the governance relationships between clinical care and research. After articulating the relationship between care and research with tissue and data, Boeckhout and Douglas stress a progressive blurring of boundary, as new epistemic objects emerge, through practices including the formatting of data, the development of new resource allocation systems, and the routinized work of patients as contributors within clinical research itself. This blurring troubles existing assumptions within dominant modes of clinical research governance that identify and further assert the clear separation of research and care. The authors further note multiplicity in the responses to the disjuncture between governance assumptions and practice, with some cases in which this hybrid form is embraced and features as a key strategy of the management, while in other cases practitioners work to purify and re-establish the separation as a workable distinction. They close by suggesting this evidences a wider trend in the complexity and uncertainty of biomedical governance that requires closer attention by all.

Brown and Williams (2015) work through Roberto Esposito’s account of biopolitics that seeks to move beyond polarised accounts of immunity and community, that can also be thought of as the private and public, in the context of cord blood banking. In doing the notion of bio-object captures the hybridity of cord blood and connects it to this more fluid notion of the blood economy. The paper daws upon sustained contact with the field, evident in interviews, focus groups, site visits, market assessments and documentary analysis. They explore the significant globalisation of cord blood distribution, through an account of the movement of tissue between Madrid’s cord blood stem cell bank and New Zealand, to demonstrate the role of cosmopolitan internationalisation in the sector. Cord blood therefore exists in a liquid immunitary regime crossing countries that destabilises traditional notions of community. A second troubling focus is the tension between the notion of cord blood given as gift and its frequent subsequent entanglement in market exchange and attribution of value and cost. Brown and Williams examine the role of the notion of waste in mediating this tension and the moral tone this sets. In doing, they show how even ‘banked’ bio-objects can be far from static through their ongoing status as potential international movers.

Tamminen (2015) focuses upon the important digital aspects of biobank operation. The work focuses upon the European Biobanking and Biomolecular Resources Research Infrastructure (BBMRI) and ongoing development of their Minimum Information About Biobank Data Sharing model (MIABIS). The model’s intended role is to facilitate the integration of European biobanks as a virtual biobank through a common standard and software interface. As such the MIABIS represents a high-profile and large scale example of a broader trend of digitally linked but geographically disparate biological collections. The paper reports ethnography conducted within the BBMRI during the process of MIABIS development. Tamminen’s account recognises biobanking as a component of big science, and the inherent data flow management issues implied in this. Firstly, the paper shows how cultural values become entangled within the work of the infrastructure. Secondly, the paper articulates governance issues at the intersection of big data and biobanking, particularly in the international context. Key to these accomplishments is the recognition of the multiple meanings of politics and the political that frame work in the area, as evident it the BBMRI’s vision and challenges, as well as the formation on novel discourses to articulate these aims and the practices used to address them. These emergent interrelations are shown to redefine the bio-objects held within the biobanks as digital, biological and social elements align in novel forms.

Stephens and Dimond (2015) explore the impacts of unanticipated happenings at a biobank, specifically focusing firstly upon significant increases in tissue submissions and secondly upon unexpected biobank closure and the subsequent redistribution of tissue. The paper reports a case study of a single anonymous biobank, here called Xbank, during the period of its closure through 14 interviews and observations over a three year period. Stephens and Dimond combine the bio-objects framework with a tissue economies approach to analyse precariousness and momentariness at Xbank and in biobanking more broadly. In doing so they deliver an unfolding narrative articulating various stages in the closure process as experienced by Xbank staff as they sought new contexts in which their tissue holdings could be stored and made valuable and available to other researchers. Key to this analysis is the relationship between value and waste, as biological material cycles though various contexts of use and under-use. The paper makes clear the physical and documentary labour necessary in creating bio-objectification, and provides an important illustration of how bio-objectification can be undone as the socio-material accomplishments that support it are either left under-maintained over an extended period of time, or swiftly disrupted for example though biobank closure.

Collectively the thematic edition uses a united theoretical framing to explore a diversity of biobanking bio-objectification processes. In doing so it presents a rich and detailed explication of the bio-objects framework in the context of a core site of biomedical innovation, socio-technical interaction, and value production. We hope to inspire further exploration of both these theoretical ideas and the social context of biobanking as these issues continue to demand inspection from policy and social science perspectives.
